# Long non-coding RNA CCHE1 modulates LDHA-mediated glycolysis and confers chemoresistance to melanoma cells

**DOI:** 10.1186/s40170-023-00309-z

**Published:** 2023-07-21

**Authors:** Zhi Ding, Junyi Yang, Baojin Wu, Yingzhi Wu, Fanli Guo

**Affiliations:** Department of Plastic Surgery, Huashan Hospital, Fudan University, Shanghai, China

**Keywords:** CCHE1, LDHA, Glycolysis, Chemoresistance, Melanoma

## Abstract

Melanoma is considered as the most common metastatic skin cancer with increasing incidence and high mortality globally. The vital roles of long non-coding RNAs (lncRNAs) in the tumorigenesis of melanoma are elucidated by emerging evidence. The lncRNA cervical carcinoma high-expressed 1 (CCHE1) was overexpressed and acted as an oncogene in a variety of cancers, while the function of CCHE1 in melanoma remains unclear. Here, we found that CCHE1 was highly expressed in melanoma and correlated with the poorer survival of melanoma patients. Depletion of CCHE1 inhibited the proliferation, induced cell apoptosis and suppressed in vivo tumor growth. To further understand the functional mechanism of CCHE1, the interacting partners of CCHE1 were identified via RNA pull-down assay followed by mass spectrometry. CCHE1 was found to bind lactate dehydrogenase A (LDHA) and acted as a scaffold to enhance the interaction of LDHA with the fibroblast growth factor receptor type 1 (FGFR1), which consequently enhanced LDHA phosphorylation and activity of LDHA. Inhibiting CCHE1 strikingly suppressed the glycolytic flux of melanoma cells and lactate generation in vivo. Further study demonstrated that CCHE1 desensitized melanoma cells to dacarbazine and inhibition of glycolysis reversed CCHE1-induced chemoresistance. These results uncovered the novel function of CCHE1 in melanoma by reprogramming the glucose metabolism via orchestrating the activity of LDHA.

**Supplementary Information:**

The online version contains supplementary material available at 10.1186/s40170-023-00309-z.

## Background

Melanoma is a common malignancy characterized by early metastasis and high mortality. Chronic UV exposure and ulcers, as well as genetic susceptibility and immunosuppression are considered as important risk factors for melanoma [1, 2]. The major treatment option for melanoma is surgical excision, and new drug approved over last 10 years also greatly improved the prognosis of melanoma [1–5]; however, due to the high tendency of recurrence and metastasis, the prognosis of patients in advanced stage remains unfavorable with the current therapies. The incidence of melanoma has been increasing globally during the past several decades, making it become a common malignancy in clinical practices [6]. Therefore, there is a pressing need to search for novel biomarkers and illustrate the molecular mechanisms that are responsible for melanoma progression.

Genome-wide sequence analysis reveals that only ~ 2% of the genome encodes proteins, while a major of the remaining transcripts are non-coding RNAs (ncRNAs) [7]. Long non-coding RNAs (lncRNAs), with the length of more than 200 nucleotides, represent a big group of ncRNAs [8]. LncRNA can physically interact with microRNAs (miRNAs), mRNAs and proteins to modulate their stability or activity [9, 10]. Researches in the last decade uncovered the important roles of lncRNAs in cancer progression, suggesting lncRNAs as potential biomarkers and therapeutic targets for cancer [11]. The cervical carcinoma high-expressed lncRNA 1 (CCHE1) was overexpressed and served as an oncogene in a variety of cancers, including cervical cancer, gastric cancer, non-small cell lung cancer and pancreatic cancer [12–16]. CCHE1 promoted the cancer development via diverse mechanisms and indicated poor prognosis of cancer patients. Silencing of CCHE1 presented strong anti-cancer efficacy, suggesting CCHE1 may be a promising target for cancer therapy. However, the function of CCHE1 in melanoma has not been fully understood.

Metabolism reprogramming has been a well-recognized hallmark of cancer [17]. To produce sufficient energy and intermediates for biosynthesis, tumor cells predominantly utilize glycolysis regardless of abundant oxygen availability, which is known as Warburg effect [18]. As a unique metabolic characteristic of cancer cells, investigating the underlying mechanisms that cooperated glycolysis and cancer cell growth could be helpful to understand the tumorigenesis of melanoma. Targeting the pathway of glycolysis might be a promising therapeutic strategy for melanoma. Multiple glycolytic enzymes are upregulated in cancers and contribute to the disease progression [19]. Lactate dehydrogenase A (LDHA) catalyzes the conversion of pyruvate to lactate, which is the last step of glycolysis and related to cancer development including melanoma [20–23]. However, the regulatory mechanism of LDHA activity and the physiological significance of LDHA inhibition in melanoma remain largely unknown.

In this study, we found that CCHE1 was overexpressed in melanoma and correlated with poor clinical outcome of melanoma. CCHE1 bound LDHA and modulated its activity in melanoma glycolysis via regulating FGFR1-mediated LDHA phosphorylation. Overexpressed CCHE1 confers chemoresistance to melanoma cells. These findings established the novel mechanism of CCHE1/FGFR1/LDHA axis in melanoma, suggesting CCHE1 as a potential target for melanoma treatment.

## Methods

### Clinical tissue samples

Fifty melanoma patients were enrolled in this study and their melanoma tissues, as well as paired adjacent non-cancer tissues (~ 3 cm away from the tumor boarder) were obtained via radical resections at the Huashan Hospital. Tissues were immediately frozen in liquid nitrogen before experiments. All the involved participants did not have other malignancies and were not received any treatments previously. To compare the expression of CCHE1 in dacarbazine-resistant or non-resistant patients, 21 patients developed complete or partial remission after two cycles of chemotherapy. These patients included 12 males and 9 females, median age 51 years (range 29–66), the ECOG (Eastern Cooperative Oncology Group) performance status 0–1 for 15 patients and 2–3 for 6 patients. In dacarbazine-failures cohort, 30 patients including 18 males and12 females, median age 54 years (range: 34–65), performance status 0–1 for 17 patients and 2–3 for 13 patients. These patients had progressive disease after a mean 3 cycles of chemotherapy. This study was performed with the approval of the Ethics Committee of Huashan Hospital. Informed consents were obtained from all the patients or their guardian.

### Cell line and transfection

Melanoma cell lines as well as the normal human melanocytes HEMn-LP cells were all purchased from the American Type Culture Collection (Manassas, VA, USA). Cells were grown in DMEM or McCoy’s 5A medium supplemented with 10% fetal bovine serum (FBS, Gibco, Gaithersburg, MD, USA) and maintained in an incubator at 37 °C with 5% CO_2_. Transfection of siRNA-control, siRNA-CCHE1, pcDNA-Flag-LDHA, or pcDNA-Flag-LDHA Y10F was performed with Polyethylenimine Linear (PEI, MW 40000, Sino Biologicals, Beijing, China) according to the manufacturer’s instructions. The cisplatin (232,120), dacarbazine (D2390), and 2-DG (D8375) were obtained from Sigma-Aldrich (St. Louis, MO, USA) and stored at 4 °C (RT). The dacarbazine resistant A375 and G-361 cells were derived from the parental cells through 2 cycles of dacarbazine treatment with lethal dose that kills 90% of cells. After the screening, cells were cultured in fresh medium with10% FBS.

### RNA extraction and RT-qPCR assay

Total RNA of tissues and cells was extracted using the RNAprep Pure Cell Kit (TIANGEN, Beijing, China). Complementary DNA (cDNA) was generated using the High Capacity cDNA Reverse-Transcription Kit (Applied Biosystems). qPCR assay was carried out to quantify CCHE1 expression using the SYBR FAST qPCR Master Mix (TIANGEN, Beijing, China) on the Bio-Rad IQ5 Real-time PCR platform (Bio-Rad, Hercules, CA, USA). CCHE1 expression was normalized to the GAPDH level. Primer sequences used in this study are shown as follows: CCHE1 forwards, 5′-AAGGTCCCAGGATACTCGC-3′; reverse, 5′-GTGTCGTGGACTGGCAAAAT-3′. Data was analyzed according to the 2^−ΔΔCT^ formula.

### Cell proliferation

Melanoma cell proliferation of melanoma cells transfected with siRNA-control or siRNA-CCHE1 was compared via the Cell Counting Kit-8 (CCK-8) assay. One hundred microliters of cell suspension (1 × 10^4^ cells/ml) were seeded into the 96-well plate and cultured overnight. After the attachment of cells to the well, cells were incubated with 10 μl of CCK-8 solution (Dojindo Laboratories, Kumamoto, Japan) at the interval of 24 h. The proliferation curve was plotted by measuring the absorbance of cells at the wavelength at 450 nm with Microplate Reader (BioTek, Winooski, VT, USA). To evaluate the chemosensitivity of melanoma cells, both A375 and G-361 cells were treated with 100 μg/ml dacarbazine or 5 μM cisplatin for 48 h, and then the cell viability was measured as described.

### Flow cytometry analysis

To measure the apoptosis of transfected melanoma cells, cells were harvested and stained with the Annexin V-fluorescein isothiocyanate (FITC)/propidium iodide (PI) for 15 min at room temperature (RT) avoid of light. The early and late apoptotic melanoma cells were detected by the flow cytometry (BD, Franklin Lakes, NJ, USA).

### Western blot

Whole cell lysates were made with NP-40 lysis buffer plus protease inhibitor (Invitrogen, Shanghai, China). Protein samples were run on the 15% SDS-PAGE and transferred onto the nitrocellulose filter membrane (GE Healthcare Life Sciences, Shanghai, Chia) using the semi-dry instrument (Bio-Rad, Hercules, CA, USA). After blocking with 5% non-fat milk in PBS for 1 h at RT, the membranes were incubated with specific primary antibodies overnight at 4 °C and HRP-conjugated secondary antibody for 2 h at RT, respectively. The protein bands were visualized using the SuperSignal West Pico Chemiluminescent Substrate Kit (Pierce, Thermo Fisher Scientific, Waltham, MA, USA) according to the manufacturer’s protocol. The signals were scanned by MYECL Imager and data analysis was performed using the ImageJ software (Version 1.6, NIH, Bethesda, MD, USA).

### Luciferase reporter assay

Melanoma cells were co-transfected with the luciferase reporter plasmids using PEI reagent. Cells were lysed after transfection for 48 h and the luciferase activity was determined by the Dual-luciferase Assay System (Promega). The luciferase activity of *Renilla* was also detected as the internal control. The experiment was performed in triplicates and data was presented from three independent repeats.

### RNA pull-down assay

pSPT19-CCHE1 vector was linearized with corresponding restriction enzymes and the full-length of CCHE1 was in vitro transcribed using the T7 RNA polymerase (Roche, Basle, Switzerland). The conjugation of biotin to CCHE1 was performed with the Biotin RNA Labeling Mix (Roche, Basle, Switzerland) according to the manufacturer’s instructions. Five micrograms in vitro transcribed biotin-CCHE1 was incubated with the whole cell lysates or his-tagged recombinant protein for 6 h at 4 °C following by precipitating with streptavidin beads (Invitrogen, Shanghai, China). The proteins that interacted with CCHE1 was detected by MS or western blot analysis.

### RNA immunoprecipitation (RIP)

Melanoma cells were lysed with the NP-40 buffer containing protease inhibitor cocktail and RNase inhibitor for 2 h at 4 °C. BCA assay was performed to determine the protein concentration. Equal amount of protein was incubated with the corresponding primary antibody or isotype IgG control at 4 °C overnight, respectively, followed by precipitated with protein A beads for 2 h. The co-precipitated RNAs were isolated using Trizol and detected by qPCR or regular PCR (CCHE1 primer, forward: 5′-TCTTCTGTCTGCTCTCGGTG-3′; reverse: 5′-CCACACCCCAATACCGTACA-3′). GAPDH was also analyzed as the internal control.

### Analysis of glucose uptake and lactate generation

Cells were incubated with 2-DG for 1 h at 37 °C. After washing three times with PBS, cells were lysed and glucose uptake was determined with the Glucose Uptake Assay kit (Abcam). For the measurement of lactate production, cells were cultured in pyruvate-free medium for 8 h in 96-well plate. The medium was collected and diluted to 6 folds with the assay buffer. The lactate levels were detected using the lactate colorimetric assay kit (Abcam) according to the manufacturer’s instructions.

### Extracellular acidification rate (ECAR) measurement

The ECAR of melanoma cells was determined using the Seahorse XFe24 Extracellular Flux Analyzer (Agilent Technologies). Cells transfected with siRNA-CCHE1 or overexpressed CCHE1 were plated in 24-well plate (20,000 cells/per well) and cultured overnight. After washing, cells were incubated with fresh assay medium and exposed to glucose (10 mM) or metabolic inhibitors including 1 μM oligomycin, and 50 mM 2-DG sequentially at the indicated time points. ECAR was detected by the Seahorse software and normalized to cell number.

### In vivo tumor growth assay

Cells were transfected with lentiviral shRNA expression vectors with the targeting sequences of CCHE1, shRNA-CCHE1#1: 5′-GGCGAGCATGTTTGTTGTTTA-3′, shRNA-CCHE1#2: 5′-GTGAGAAATGAGCGGATTACC-3′ as previously reported [24]. A non-targeting shRNA 5′-CCTAAGGTTAAGTCGCCCTCG-3′ was used as the scramble control. In briefly, these sequences were constructed into the lentivirus vector backbone pLKO.1-puro (Addgene ID#10,879). For producing lentiviral particles, 293 T cells were transiently co-transfected with the pLKO.1 shRNA plasmid, the packaging plasmid psPAX2 (Addgene ID#12,260) and the envelope plasmid pMD2.G (Addgene ID#12,259) at the ratio of 4:3:1. Stable A375 cells were obtained by lentivirus infection after puromycin selection and injected subcutaneously into the flanks of 5-week old female BALB/c nude mouse (*n* = 6, Charles River). Tumor growth was measured every 3 days using calipers and tumor volume was calculated according to the formula *V* = (length × width^2^)/2. After 20 days, mice were anesthetized and the tumor weight were measured. Similarly, to measure chemo-induced tumor growth inhibition, 3 million A375 cells with lentiviral vector expressing vehicle or CCHE1 were subcutaneously implanted. When tumor volume reached around 150 mm^3^, dacarbazine (100 mg/kg) or PBS was intraperitoneally injected every other day for 2 weeks. Tumor volume was measured as described. Mice were sacrificed when tumor volume up to 2000 mm^3^. This experiment was approved by the Ethics Committee of Huashan Hospital.

### Statistical analysis

Data were obtained from three independent experiments and shown as mean ± standard deviation. Student’s *t* test or one-way analysis of variance (ANOVA) followed by Tukey test was performed for the statistical analysis of two groups or multiple groups using the GraphPad Prism 7.0. The correlation between CCHE1 and the prognosis of melanoma patients was assessed via the log-rank test. *P* < 0.05 was defined as statistical significance. *p < 0.05, **p < 0.01, ***p < 0.001.

## Results

### CCHE1 was overexpressed in melanoma and correlated with the poor prognosis of melanoma

**Fig. 1:**
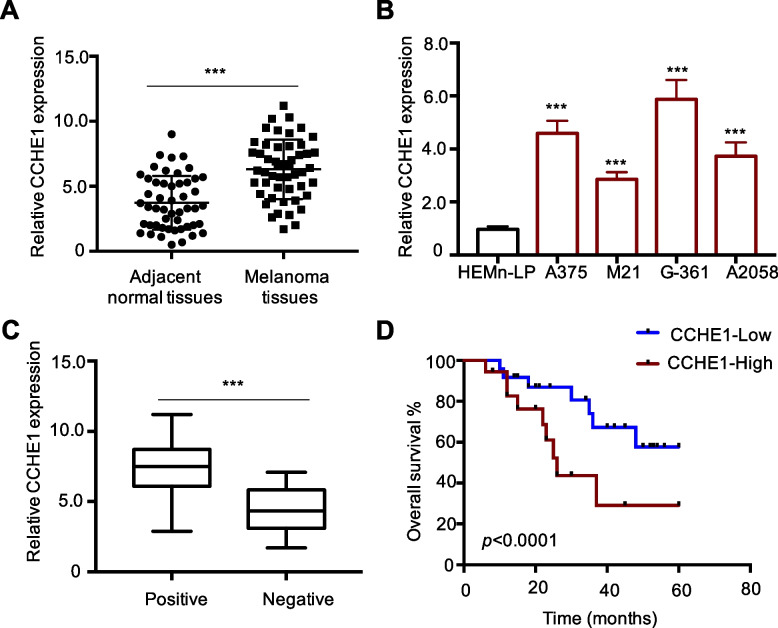
CCHE1 is overexpressed and correlated with melanoma prognosis. **A** qPCR analysis of CCHE1 expression in melanoma tumor tissues and paired non-cancerous tissues. **B** CCHE1 expression in melanoma cells (A375, M21, G-361, and A2058) and normal melanocytes was detected and higher levels of CCHE1 were observed in melanoma cell lines. **C** qPCR determination of CCHE1 levels in melanoma patients with or without lymph node metastasis. **D** Melanoma patients were divided into CCHE1-high or low group according to the median expression value of CCHE1. Kaplan–Meier method and log-rank test revealed that high CCHE1 expression was correlated with the short overall survival of melanoma patients. The statistical significance was determined by the log-rank test

To assess whether CCHE1 plays a role in melanoma, RT-qPCR was performed with 50 pairs of melanoma tumor tissues and matched non-cancerous tissues. As indicated in Fig. 1A, compared with the controls, CCHE1 expression levels were significantly increased in melanoma samples. The expression of CCHE1 in a panel of melanoma cells and normal HEMn-LP melanocytes was also compared. Upregulated CCHE1 levels were found in melanoma cell lines in contrast to the normal melanocytes (Fig. 1B). The correlation analysis for CCHE1 expression and melanoma progression showed that melanoma patients in advanced stage carried relative higher abundance of CCHE1 in comparison with those free of lymph node metastasis (Fig. 1C). To provide more evidence to highlight the clinical value of CCHE1 in melanoma, all the enrolled patients were divided into CCHE1 high or low groups according to the median expression value of CCHE1. The Kaplan–Meier survival curve indicated that higher CCHE1 expression was remarkably correlated with the unfavorable overall survival of melanoma patients (Fig. 1D). All these results suggested the potential involvement of CCHE1 in melanoma progression.

### CCHE1 was essential for melanoma cell growth

**Fig. 2:**
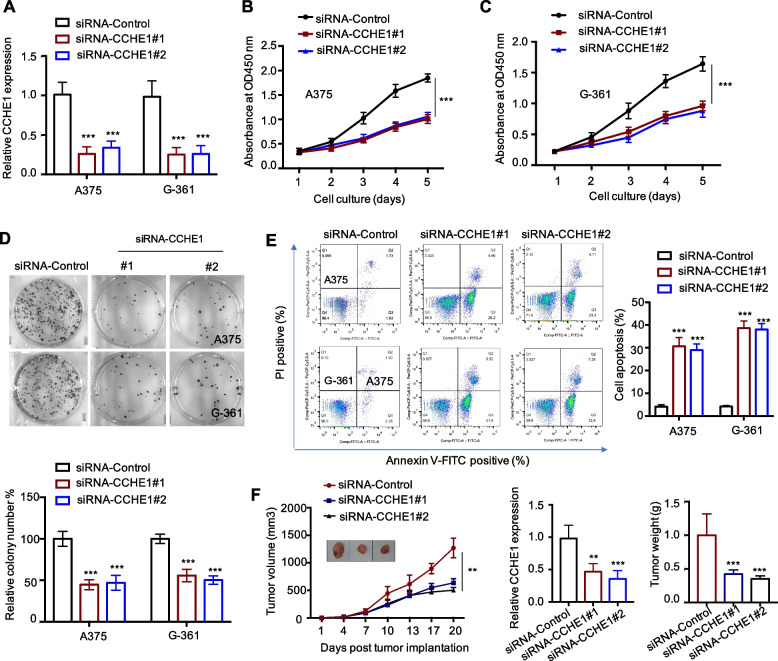
CCHE1 was essential for melanoma cell growth. **A** Melanoma cells were transfected with siRNA-control or siRNA-CCHE1, and CCHE1 knockdown by siRNA was confirmed by qPCR. **B**, **C** CCK-8 assay was performed to compare the proliferation of melanoma cells expressing siRNA-control or siRNA-CCHE1. Reduced proliferation of melanoma cells with CCHE1 depletion detected by CCK-8 assay. **D** Clone formation assay of melanoma cells transduced with siRNA-CCHE1 was significantly inhibited. **E** Flow cytometry analysis showed that CCHE1 knockdown triggered melanoma cell apoptosis compared with the control cells. **F** A375 xenograft mouse model was established with stable cells expressing shRNA-control or shRNA-CCHE1. The tumor volume was measured twice a week. CCHE1 depletion significantly delayed the tumor growth (left panel) and representative tumors were shown. The knockdown efficacy of CCHE1 was confirmed by RT-qPCR analysis at the end of the study (middle panel). Tumor weight was weighted (right panel). **p < 0.01, ***p < 0.001 *vs*. shRNA-control

To validate the cancer-promoting effects of CCHE1 in melanoma, the cellular behaviors of melanoma cells were determined followed by CCHE1 depletion. The knockdown efficacy of siRNA-CCHE1 in A375 and G-361 cells was validated by RT-qPCR as indicated in Fig. 2A. The CCK-8 assay showed that compared with cells expressing siRNA-control, CCHE1 depletion strikingly inhibited the melanoma cell proliferation (Fig. 2B, C). The clone formation capacity of melanoma cells was significantly reduced upon CCHE1 knockdown (Fig. 2D). The effects of CCHE1 knockdown on melanoma cell growth were also determined by analyzing the cell apoptosis. The result showed that CCHE1 depletion remarkably increased both the early and late apoptosis of melanoma cells (Fig. 2E). To further evaluate the essential role of CCHE1 in the tumorigenesis of melanoma, in vivo cell-based xenograft tumor model was established with A375 cells that stably expressed the lentiviral shRNA vectors targeting CCHE1 or scramble control. As shown in Fig. 2F, CCHE1 knockdown significantly repressed the tumor growth, which was consistent with the in vitro data. The downregulation of CCHE1 in tumors at the endpoint was validated by RT-qPCR. Collectively, these data demonstrated that CCHE1 was functionally important in regulating melanoma cell growth.

### CCHE1 interacted with and regulated the activity of LDHA

**Fig. 3:**
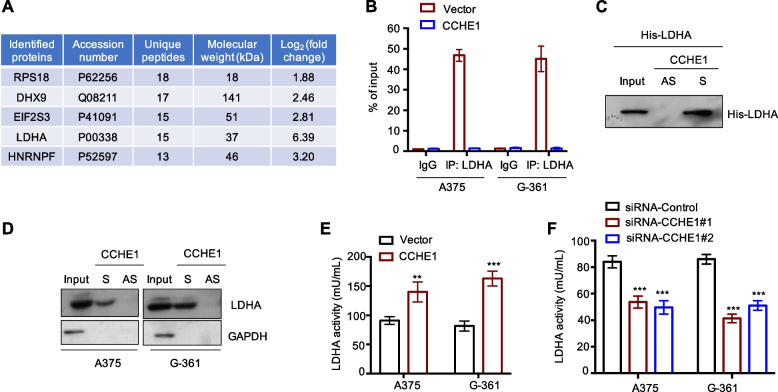
CCHE1 interacted with LDHA and regulated LDHA activity. **A** partial list of proteins identified by mass spectrometry that specially bound CCHE1. The gene name, molecular weight, number of unique peptides, and log_2_ fold change were shown. **B** RIP assay was performed using anti-LDHA antibody and significantly enriched CCHE1 abundance was found, demonstrating the specific binding CCHE1 with LDHA in melanoma cells. **C**, **D** His-tagged recombinant LDHA (**C**) or LDHA in whole cell lysates (**D**) was pull-down by biotin-labeled CCHE1 but not the antisense CCHE1. **E**, **F** LDHA activity was determined in melanoma cells with CCHE1 overexpression (**E**) or knock-down (**F**) using the lactate dehydrogenase activity assay kit. Overexpressed CCHE1 significantly increased the LDHA activity (**E**), while CCHE1 depletion reduced the activity of LDHA (**F**). The signals were normalized to cell numbers. AS, antisense; S: sense. ***p < 0.001 *vs*. control

To understand the functional mechanism of CCHE1 in melanoma tumorigenesis, RNA pull-down assay was performed with CCHE1 followed by mass spectrometry to identify CCHE1 associated proteins. As a result, 43 proteins were detected with log_2_ fold change (CCHE1/control) > 1.50 and *p* value < 0.05 (Supplementary Table S1). The top 5 identified proteins, which were ranked according to the number of unique peptides were shown in Fig. 3A. Notably, LDHA was found as one of the top putative binding proteins of CCHE1 (Fig. 3A). LDHA catalyzes the conversion of pyruvate to lactate, the last step of the glycolysis. The interaction of CCHE1 with LDHA was validated by RNA immunoprecipitation (RIP) assay using anti-LDHA antibody. As indicated in Fig. 3B, significant enrichment of CCHE1 was present in LDHA immunoprecipitates compared with the IgG control. Additionally, the binding between LDHA and CCHE1 was further validated by RNA pull down assay. Biotin labeled CCHE1 or antisense CCHE1 was incubated with His-LDHA, the binding of LDHA with CCHE1 was detected by precipitating with streptavidin beads. Western blot showed the strong signal of LDHA within the precipitation samples but not the antisense control (Fig. 3C). Consistently, this finding was also validated by the in vitro pull-down experiment by incubating biotin-CCHE1 with the lysates of A375 and G-361 cells (Fig. 3D). These observations demonstrated the binding between CCHE1 and LDHA. To further explore whether the binding of CCHE1 affected the activity of LDHA, melanoma cells were overexpressed with CCHE1, and the LDHA activity was measured. As indicated in Fig. 3E, activity of LDHA was enhanced upon CCHE1 transfection. Consistently, knockdown of CCHE1 reduced the LDHA activity (Fig. 3F). Collectively, these results demonstrated that CCHE1 interacted with LDHA and modulated its activity in melanoma cells.

### CCHE1 promotes aerobic glycolysis of melanoma cells

**Fig. 4:**
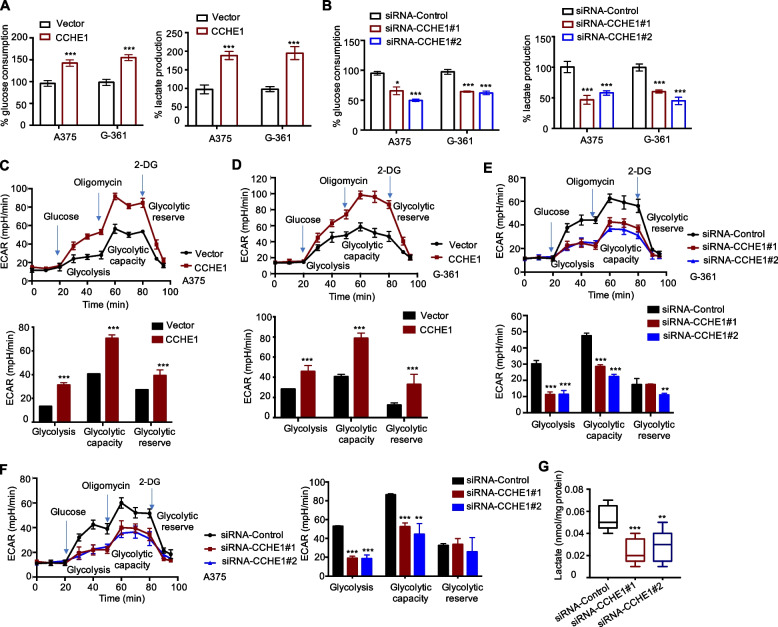
CCHE1 promoted melanoma cell glycolysis. **A** CCHE1 overexpression promoted the glucose uptake (left panel) and lactate levels (right panel). **B** CCHE1 depletion significantly reduced both the glucose uptake (left panel) and lactate production (right panel) of melanoma cells compared with the control cells. **C**, **D** Melanoma cells were transfected with control vector of CCHE1. The glycolysis fulx was determined by detecting the EACR using the Seahorse analyzer. 10 mM glucose, 1 μM ATP synthase inhibitor oligomycin, and 50 mM 2-DG was added at the indicated time points. The values of glycolysis, glycolytic capacity and glycolytic reserve were calculated by Seahorse XF24 software. Significantly enhanced glycolysis flux was observed with CCHE1 overexpression. **E**, **F** CCHE1 knockdown inhibited the glycolysis fulx both in A375 and G-361 cells. In C-F, data was obtained from three independent experiments and statistical analysis was performed via the two-sided Student’s* t* test. **G** The tumors of A375 xenograft mouse model were isolated at the end of the experiment and the lactate levels were detected. The lactate levels of tumors carrying CCHE1 depletion ware lower than that of the shRNA-control group. Data was normalized to protein concentrations. * *p* < 0.05, ** p < 0.01, ***p < 0.001 *vs*. control

Considering the important role of LDHA in aerobic glycolysis, the interaction of CCHE1 with LDHA might affect the glycolysis of melanoma cells. To test this hypothesis, the glucose consumption and lactate generation were measured with CCHE1 overexpression or depletion. As indicated in Fig. 4A, CCHE1 overexpression significantly upregulated the glucose uptake and lactate production in both A375 and G-361 cells. Consistently, the glucose consumption and lactate generation were reduced following CCHE1 depletion (Fig. 4B). To provide more evidence to evaluate the key role of CCHE1 in glycolysis, the glycolytic flux was measured using the Seahorse Analyzer to quantify the extracellular acidification rate (ECAR). The data showed that CCHE1 overexpression strikingly increased the overall glycolytic flux including glycolysis, glycolytic reserve and glycolytic capacity of melanoma cells (Fig. 4C, D). Meanwhile, depletion of CCHE1 in A375 and G-361 cells inhibited the glycolytic flux compared with the control cells (Fig. 4E, F). These finding demonstrated the positive regulation of CCHE1 in the aerobic glycolysis of melanoma. To further support this conclusion, the lactate levels of tumors from the in vivo study shown in Fig. 2F were determined. Consistent with the in vitro data, less lactate was detected in tumor with CCHE1 depletion (Fig. 4G). These results provided strong evidence to reveal the key regulatory function of CCHE1 in the aerobic glycolysis of melanoma.

### CCHE1 regulated LDHA phosphorylation via binding FGFR1

**Fig. 5:**
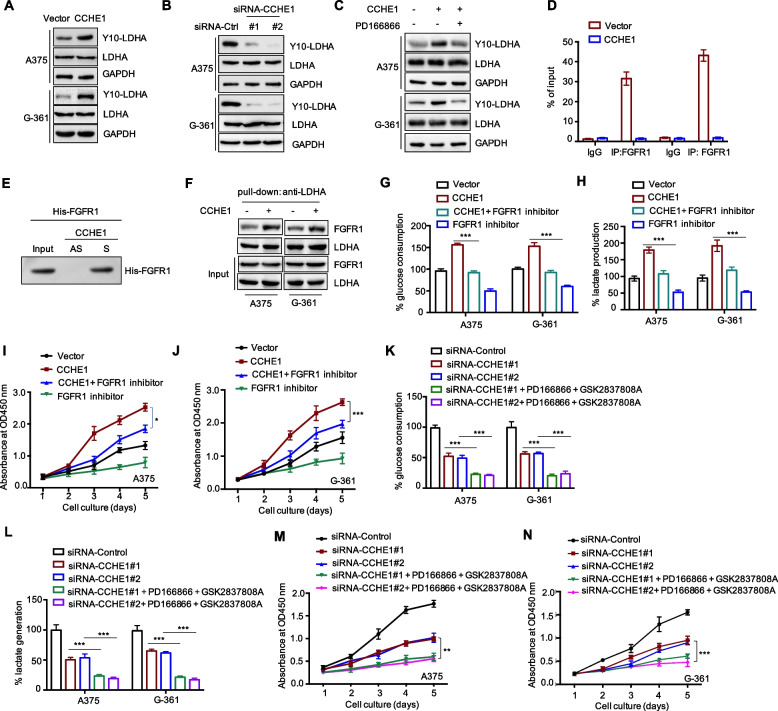
CCHE1 modulated LDHA phosphorylation via FGFR1. **A** CCHE1 was overexpressed in melanoma cells and the level of phos-LDHA (Y10) was detected by western blot. Enhanced LDHA-Y10 was observed with CCHE1 overexpression. **B** The phos-LDHA (Y10) was decreased upon CCHE1 depletion in both A375 and G-361 cells. (C) Melanoma cells were treated with 2.5 μM FGFR1 inhibitor PD166866 for 24 h, and the p-LDHA (Y10) was determined via western blot. Blocking FGFR1 attenuated CCHE1-promoted LDHA-Y10 phosphorylation. **D** The specific binding between CCHE1 and FGFR1 was determined by the RIP assay, which showed the significant enrichment of CCHE1 in the immunoprecipitates with anti-FGFR1 antibody. **E** Pull-down assay was performed with his tagged recombinant FGFR1 protein and biotin-labeled CCHE1. FGFR1 interacted with CCHE1 but not the antisense CCHE1. **F** Cells were transfected with control vector or CCHE1, and the interaction between LDHA and FGFR1 was detected by co-IP assay using anti-LDHA antibody. Enhanced abundance of FGFR1 was found in the precipitates of LDHA with CCHE1 overexpression. **G**, **H** Blockade of FGFR1 significantly inhibited CCHE1-promoted glucose uptake and lactate generation. **I**, **J** FGFR1 inhibition weaken the positive role of CCHE1 in melanoma cell proliferation. **K**, **L** Both the glucose consumption and lactate production were significantly reduced with the addition of inhibitors of FGFR1 (PD166866, 2.5 μM) and LDHA (GSK2837808A, 10 μM) in CCHE1-depleted melanoma cells. **M**, **N** Depletion of CCHE1 combined with inhibition of FGFR1 and LDHA obviously suppressed the proliferation of melanoma cells. *p < 0.05, **p < 0.01, ***p < 0.001

It is well established that LDHA activity is fine-tuned by its phosphorylation at Y10 amino acid. To evaluate whether Y10 phosphorylation was involved in the function of CCHE1 in glycolysis, the LDHA Y10-phosphorylation level was detected in cells with CCHE1 overexpression. As indicated in Fig. 5A, CCHE1 overexpression remarkably enhanced the LDHA phosphorylation level in contrast to cells expressing control vector. Consistently, CCHE1 depletion significantly reduced the phosphorylation of Y10 amino acid of LDHA (Fig. 5B). Down-regulation of p-LDHA Y10 by CCHE1 knockdown was also validated in the in vivo xenografts (Supplementary Figure S1A). Phosphorylation of LDHA was reported to be dominantly regulated by the fibroblast growth factor receptor type 1 (FGFR1), which modulated the enzymatic activity of LDHA in glycolysis [25]. In the mass spectrometry analysis, FGFR1 was found as a CCHE1-binding protein candidate (Supplementary Table S1). To further understand whether CCHE1 modulated LDHA phosphorylation via regulating FGFR1, melanoma cells were treated with FGFR1 inhibitor PD-166866 and the data showed that, the enhanced LDHA phosphorylation by CCHE1 was abolished in the presence of PD-166866 (Fig. 5C). The interaction between CCHE1 and FGFR1 was further confirmed by the RIP assay, which showed that CCHE1 was significantly enriched in FGFR1 immunoprecipitates compared with that of the normal control IgG (Fig. 5D). The direct interaction of CCHE1 with FGFR1 was also confirmed by in vitro pull-down assay with his tagged recombinant FGFR1 (Fig. 5E). To determine whether the increased phosphorylation level of LDHA was achieved by enhancing the interaction between LDHA and FGFR1, co-immunoprecipitation (co-IP) was performed with anti-LDHA antibody and the data showed that CCHE1 overexpression significantly promoted the binding between LDHA and FGFR1 (Fig. 5F). Consistently, the interaction between FGFR1 and LDHA was also assessed in CCHE1-depleted cells. The data showed that depletion of CCHE1 attenuated the binding of FGFR1 with LDHA (Supplementary Figure S1B).

To confirm whether the function of CCHE1 in melanoma glycolysis was mediated via FGFR1/LDHA axis, cells were co-transfected with CCHE1 in the presence of FGFR1 inhibitor. As shown in Fig. 5G, H, blockade of FGFR1 significantly inhibited the promoting function of CCHE1 in the glucose uptake and lactate generation. Consistent with these results, the CCK8 assay indicated that overexpression of CCHE1 could not promote the cell proliferation in the presence of FGFR1 inhibitor (Fig. 5I, J). These results demonstrated FGFR1 involved in the function of CCHE1 in melanoma. To demonstrate the key function of CCHE1/FGFR1/LDHA regulatory axis in melanoma, cells expressing siRNA-CCHE1 were treated with the inhibitors of FGFR1 and LDHA, and the cell glycolysis was detected. The data showed that combination of FGFR1-LDHA inhibitors could further suppress the glycolysis of melanoma cells in CCHE1-depleted cells (Fig. 5K, L). Additionally, the proliferation of melanoma cells that were co-treated with inhibitor of FGFR1 PD166866 and LDHA inhibitor GSK2837808A was detected by CCK-8 assay. Significantly reduced cell proliferation was observed with the co-inhibition of FGFR1 and LDHA in CCHE1-depleted melanoma cells (Fig. 5M, N). Additionally, rescue experiments were also performed to prove LDHA Y10 phosphorylation was the downstream of CCHE1. In detail, plasmids expressing LDHA or LDHA Y10F mutant with Flag tag were transfected into the CCHE1 knockdown A375 and G-361 cells. Overexpression of LDHA or LDHA Y10F was validated by western blot with anti-Flag antibody as shown in Supplementary Fig. 2A. The CCK-8 assay showed that co-transfection of LDHA but not LDHA Y10F could rescue the reduced proliferation of melanoma cells by CCHE1 depletion (Supplementary Figure S2B). Additionally, the ECAR analysis also indicated that LDHA not LDHA Y10F transfection significantly increased the ECAR of melanoma cells compared with the LDHA knockdown group (Supplementary Figure S2C, D). These results suggested that phosphorylation of LDHA at the tyrosine 10 plays a key role in mediating the function of CCHE1 in melanoma.

### CCHE1 desensitized melanoma cells to dacarbazine via glycolysis

**Fig. 6:**
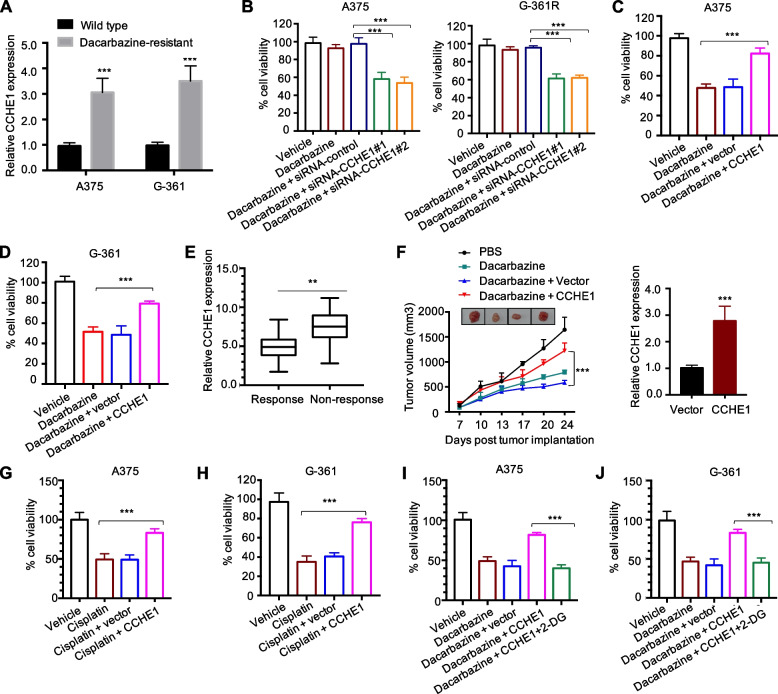
CCHE1 desensitized melanoma cells to dacarbazine via glycolysis. **A** CCHE1 expression was significantly increased in dacarbazine-resistant melanoma cells. **B** Dacarbazine resistant A375 (A375R) and G-361 (G-361R) cells were transfected with siRNA-control or siRNA-CCHE1. Cells were treated with dacarbazine for 48 h and the cell viability was determined by CCK-8 assay. **C**, **D** Cells were treated with 100 μg/ml dacarbazine for 48 h, and CCHE1 overexpression significantly reversed dacarbazine-induced cell death. **E** The expression of CCHE1 in dacarbazine resistant melanoma patients was significantly higher than patients sensitive to dacarbazine. **F** In vivo xenograft mouse model was established by transplanting A375 cells transfected with lentivirus expressing control vector or CCHE1. Mice were treated with dacarbazine and the tumor volume of the CCHE1 group was significantly increased compared with the control after dacarbazine exposure. Overexpression of CCHE1 in the xenograft tumors was validated at the endpoint. Representative tumors were shown. **G**, **H** Transfection of CCHE1 significantly reduced the sensitivity of melanoma cells to cisplatin treatment. **I**, **J** Melanoma cells were treated with dacarbazine and overexpression of CCHE1 increased the cell viability. Addition of 2-DG attenuated the function of CCHE1 in desensitized melanoma cells to dacarbazine. *p < 0.05, **p < 0.01, ***p < 0.001

Chemotherapy using dacarbazine combined with surgical resection remain one of the standard treatment options for melanoma. However, limited clinical response rate was found as a consequence of chemotherapy failure. In this study, we found that CCHE1 expression was remarkably increased in dacarbazine-resistant A375 (A375 R) and G-361 (G-361R) than their parental cells (Fig. 6A). To explore whether CCHE1 regulated the dacarbazine resistance, A375R and G-361R cells were transfected with siRNA-control or siRNA-CCHE1. The results showed that depletion of CCHE1 significantly reduced the resistance of A375R and G-361R cells to dacarbazine (Fig. 6B). This finding suggested the possible role of CCHE1 in melanoma chemoresistance. To further support this hypothesis, cells were transfected with CCHE1 or scramble vector and incubated with dacarbazine. The cell viability analysis showed that CCHE1 overexpression significantly reduced the sensitivity of melanoma cells to dacarbazine (Fig. 6C, D). Additionally, analysis of the tissue samples revealed that patients sensitive to chemotherapeutic drugs carrying low CCHE1 expression, and individuals resistant to chemotherapy showing high CCHE1 levels (Fig. 6E).

To provide more evidence for the function of CCHE1 in melanoma chemoresistance, in vivo xenograft mouse model was established by transplanting A375 cells that stably expressed control vector or CCHE1. Mice were treated with dacarbazine and the tumor volume of the CCHE1 group was significantly increased compared with the control after dacarbazine exposure (Fig. 6F). Overexpression of CCHE1 in the xenograft tumors was validated at the endpoint (Fig. 6F, right panel). This result demonstrated that highly expressed CCHE1 desensitized melanoma cells to dacarbazine treatment. Additionally, the chemoresistance of CCHE1 in melanoma was also confirmed by treating cells with cisplatin. As shown in Fig. 6G, H, the cell viability after cisplatin treatment was obviously higher with the transfection of CCHE1 compared with cells carrying control vector. To further demonstrate whether CCHE1-induced chemoresistance of melanoma cells was through enhancing glucose metabolism, cells were incubated with the 2-DG as the competitive glycolytic inhibitor. The CCK8 assay showed that inhibition of glycolysis reversed CCHE1-induced chemoresistance of melanoma cells (Fig. 6I, J). These data demonstrated that CCHE1 modulated the glycolysis and confers chemoresistance to melanoma cells.

## Discussion

The initiation and development of melanoma requires a series of oncogenic stimuli or loss-function of tumor suppressors. The critical involvement of lncRNA in melanoma was proved by recent studies through regulating oncogenic or tumor suppressive pathways [26–28]. Diverse mechanisms of lncRNA in cancer including regulating transcription, chromatin modification, pre-mRNA splicing, and microRNA sequestration have been reported [28], which indicates targeting lncRNA might be a therapeutic approach for melanoma. Here, we identified CCHE1 as an oncogenic regulator of aggressive melanoma (Fig. 6G). CCHE1 was highly expressed in melanoma and associated with the poorer survival of melanoma patients, suggesting the potential of CCHE1 in the risk prognostication of melanoma.

CCHE1 was a newly identified lncRNA, acting as an oncogene in a variety of carcinomas via regulating the cell proliferation, apoptosis, invasion, and differentiation [12–16]. Recent study revealed that CCHE1 promoted the tumorigenesis of hepatocellular carcinoma through ERK/MAPK signaling [29]. The oncogenic function of CCHE1 was also established in osteosarcoma, where CCHE1 interacted with ROCK1 and facilitated cancer cell invasion [30]. Additionally, CCHE1 accelerated the progression of oral squamous cell carcinoma by sponging the function of miR-922 [31]. All these findings indicated CCHE1 as a key player in the development of multiple cancers. In this study, CCHE1 depletion inhibited the proliferation, and induced apoptosis of melanoma cells. The critical involvement of CCHE1 in melanoma was also demonstrated by in vivo study, where knockdown of CCHE1 significantly delayed the tumor growth. These evidences suggested the possibility that inhibits melanoma progression via targeting CCHE1. The pan-cancer inhibitory potency with CCHE1 depletion calls for more deep studies.

Metabolism reprogramming is a hallmark of cancer, which means cancer cells preferentially catalyze glucose metabolism via aerobic glycolysis to supply enough intermediates to sustain the rapid growth of cancer cells [17, 32]. As a unique characteristic of malignancy, targeting the glycolysis pathway has been a powerful tool to interrupt cancer cell growth. Among all the enzymes involved in glycolysis, LDHA catalyzes the reversible conversion of pyruvate to lactate, which is also the last step of glycolysis and positively correlated with the efficacy of Warburg effect [33, 34]. LDHA inhibitors presented as a promising therapeutic option, as blocking the function of LDHA leads to cancer cell apoptosis. The activity of LDHA was modulated via multiple mechanisms. Notably, post-translational modifications including phosphorylation, acetylation play a key role in modulating the activation of LDHA [35, 36]. Among them, Y10 phosphorylation of LDHA by intracellular kinase FGFR1 enhanced LDHA activity and provided pro-metastatic advantage to cancer cells [37, 38]. Therefore, LDHA phosphorylation is emerging as a potential drug target to disrupt cancer progression. In the study, we found that CCHE1 acted as a scaffold to facilitate the binding between FGFR1 and LDHA, which enhanced LDHA activity and glycolysis of melanoma cells. Significantly reduced melanoma cell proliferation and glycolysis were observed with co-inhibition of FGFR1 and LDHA in CCHE1-depleted cells, suggesting the key function of CCHE1/FGFR1/LDHA regulatory axis in melanoma. The therapeutic potentials of targeting CCHE1 and co-inhibiting FGFR1/LDHA in melanoma deserve more investigation by the in vivo studies. FGFR1 was highly expressed in a majority of melanoma cases, which created the signals in the tumor microenvironment to accelerate angiogenesis, cell growth, and therapeutic resistance [39–41]. As a direct binding partner of FGFR1, CCHE1 was a novel regulator of glycolysis and also shed light on a new avenue for disrupting melanoma cell growth.

The treatment of melanoma remains unsatisfied by its broad range of chemoresistance [42–44]. Cisplatin and dacarbazine are the mostly used chemotherapy agents against melanoma, which kills cancer cells via triggering DNA damage and cell apoptosis. Understanding the mechanism of melanoma chemoresistance and its genetic heterogeneity will benefit the design of new therapeutic options. In this study, CCHE1 was found to be overexpressed in dacarbazine-resistant melanoma cells. CCHE1 overexpression confers chemoresistance to melanoma. Consistent with the function of CCHE1 in glycolysis, blockade of glucose metabolism reversed CCHE1-induced melanoma resistance to dacarbazine. These results suggested that combined therapies by targeting CCHE1 may be the optimal strategy to prevent the melanoma chemoresistance.

## Conclusions

Our results demonstrated the overexpression of CCHE1 in melanoma and was correlated with the poorer survival of melanoma patients. CCHE1 promoted the glycolysis and proliferation of melanoma cells by enhancing FGFR1-mediated LDHA phosphorylation and activation. CCHE1 overexpression confers chemoresistance to melanoma. Depletion of CCHE1 inhibited melanoma cell growth both in vitro and in vivo, suggesting RNA-interference-based strategies that targets CCHE1 for melanoma treatment.

## Supplementary Information

A**dditional file 1: Supplementary Figure S1.** CCHE1 regulates the phosphorylation of LDHA at Y10.The phosphorylation levels of LDHA Y10 in the xenograft tumors carrying shRNA-control or shRNA-CCHE1 were detected at the end of the in vivo study. A375 and G-361 were transfected with shRNA-control or siRNA-CCHE1. The interaction between FGFR1 and LDHA was explored by pull-down assay using anti-LDHA antibody.

**Additional file 2: Supplementary Figure S2.** The phosphorylation of LDHA Y10 mediated the function of CCHE1 in the proliferation and glycolysis of melanoma cell. Overexpression of Flag-LDHA or FLAG-LDHA Y10F mutant was detected by western blot with anti-Flag antibody.

**Additional file 3: Supplementary Table S1.** CCHE1-interesting proteins>1.50 and *p*<0.05) identified by MS.

**Additional file 4.**

## Data Availability

All data generated or analyzed in this study are included in the manuscript.

## Abbreviations

CCHE1: Cervical carcinoma high-expressed lncRNA 1
LDHA: Lactate dehydrogenase A
FGFR1: Fibroblast growth factor receptor type 1
lncRNAs: Long non-coding RNAs
miRNAs: MicroRNAs
RIP: RNA immunoprecipitation
