# Supplementary figures and images for: Long non-coding RNA CCHE1 modulates LDHA-mediated glycolysis and confers chemoresistance to melanoma cells
Source: Cancer Metab. 2023 Jul 21;11:10. doi: 10.1186/s40170-023-00309-z (PMC10360318; doi:10.1186/s40170-023-00309-z)

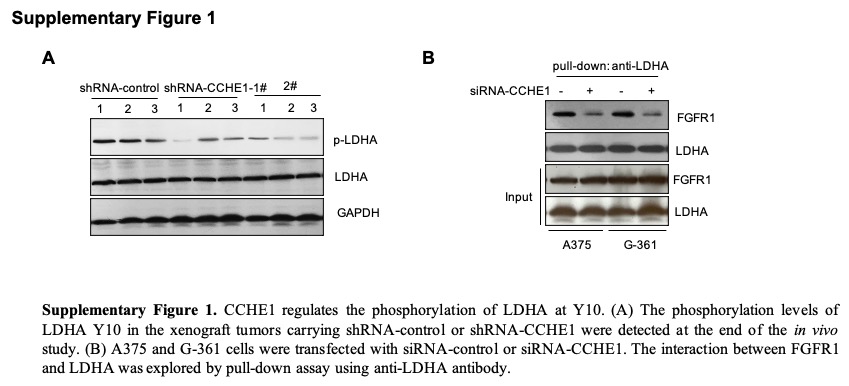

Supplement: Supplementary file 1 — Additional file 1: Supplementary Figure S1. CCHE1 regulates the phosphorylation of LDHA at Y10.The phosphorylation levels of LDHA Y10 in the xenograft tumors carrying shRNA-control or shRNA-CCHE1 were detected at the end of the in vivo study. A375 and G-361 were transfected with shRNA-control or siRNA-CCHE1. The interaction between FGFR1 and LDHA was explored by pull-down assay using anti-LDHA antibody. [file 40170_2023_309_MOESM1_ESM.jpg]

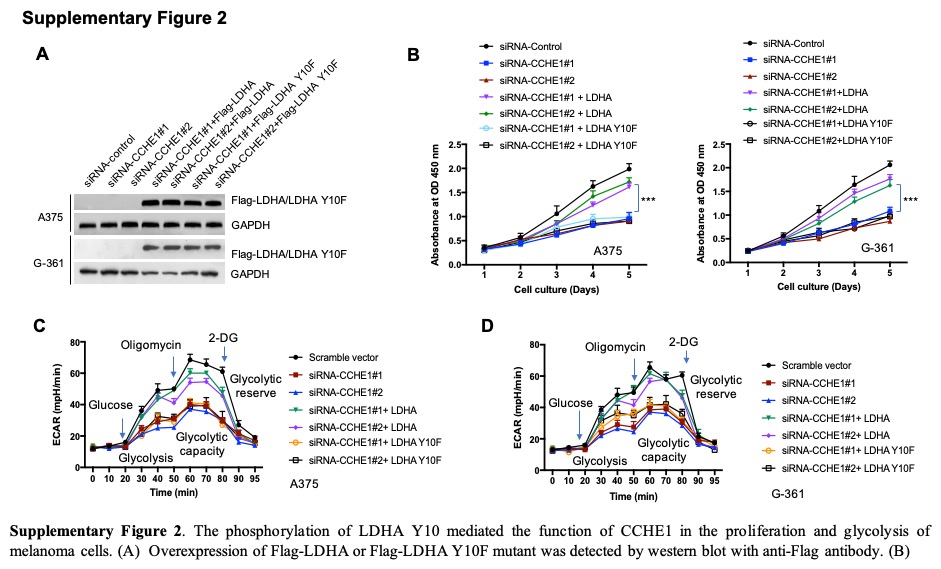

Supplement: Supplementary file 2 — Additional file 2: Supplementary Figure S2. The phosphorylation of LDHA Y10 mediated the function of CCHE1 in the proliferation and glycolysis of melanoma cell. Overexpression of Flag-LDHA or FLAG-LDHA Y10F mutant was detected by western blot with anti-Flag antibody. [file 40170_2023_309_MOESM2_ESM.jpg]

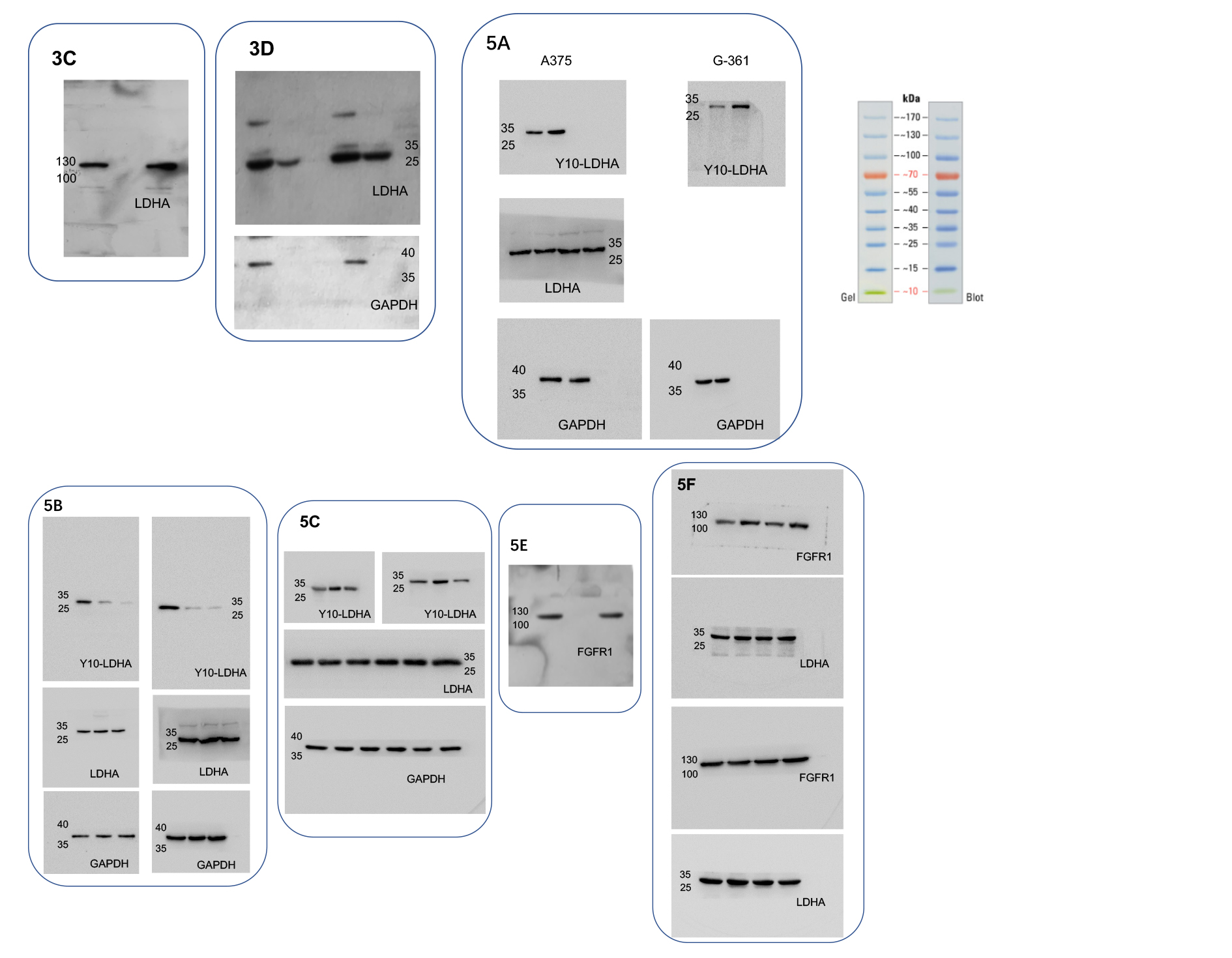

Supplement: Supplementary file 4 — Additional file 4. [file 40170_2023_309_MOESM4_ESM.jpg]
