# Supplementary material for: Long non-coding RNA CCHE1 modulates LDHA-mediated glycolysis and confers chemoresistance to melanoma cells
Source: Cancer Metab. 2023 Jul 21;11:10. doi: 10.1186/s40170-023-00309-z (PMC10360318; doi:10.1186/s40170-023-00309-z)
Supplement: Supplementary file 3 — Additional file 3: Supplementary Table S1. CCHE1-interesting proteins>1.50 and p<0.05) identified by MS. [file 40170_2023_309_MOESM3_ESM.docx]

**Supplementary Table 1.** CCHE1-interesting proteins (log_2_ (fold change)>1.50 and p<0.05) identified by MS

|  | Identified proteins | Accession number | Unique peptides | Molecular weight (KDa) | Log_2_ (fold change) |
| --- | --- | --- | --- | --- | --- |
| 1 | RPS18 | P62256 | 18 | 18 | 1.88 |
| 2 | DHX9 | Q08211 | 17 | 141 | 2.46 |
| 3 | EIF2S3 | P41091 | 15 | 51 | 2.81 |
| 4 | LDHA | P00338 | 15 | 37 | 6.39 |
| 5 | HNRNPF | P52597 | 13 | 46 | 3.20 |
| 6 | HNRNPM | P52272 | 13 | 78 | 4.38 |
| 7 | ZNF90 | A0A087WZ72 | 10 | 14 | 1.52 |
| 8 | STRAP | Q9Y3F4 | 10 | 38 | 2.46 |
| 9 | ROCK1 | Q13464 | 8 | 158 | 3.04 |
| 10 | H2A | Q14204 | 7 | 14 | 6.62 |
| 11 | FGFR1 | P11362 | 7 | 92 | 3.18 |
| 12 | ETFA | P13804 | 7 | 35 | 2.74 |
| 13 | LAMB3 | Q13751 | 6 | 130 | 1.80 |
| 14 | TGM2 | P21980 | 6 | 77 | 2.35 |
| 15 | USP11 | P51784 | 5 | 110 | 3.30 |
| 16 | KIF3A | Q9Y496 | 5 | 80 | 3.19 |
| 17 | DDX24 | Q9GZR7 | 4 | 96 | 2.52 |
| 18 | ANAPC5 | Q9UJX4 | 4 | 85 | 1.94 |
| 19 | HDAC3 | 015379 | 4 | 49 | 2.14 |
| 20 | RAD18 | Q9NS91 | 4 | 56 | 2.00 |
| 21 | TARDBP | Q13148 | 4 | 45 | 3.26 |
| 22 | HSP7C | P11142 | 3 | 71 | 1.58 |
| 23 | SLC3A2 | P08195 | 3 | 68 | 1.92 |
| 24 | PDHA1 | P08559 | 3 | 43 | 2.08 |
| 25 | AK6 | Q9Y3D8 | 3 | 20 | 1.67 |
| 26 | MLH1 | P40692 | 3 | 85 | 3.50 |
| 27 | VDAC1 | P21796 | 3 | 31 | 2.25 |
| 28 | HNRNPL | P14866 | 3 | 64 | 1.62 |
| 29 | CDK9 | P50750 | 3 | 43 | 2.31 |
| 30 | RPL35 | P42766 | 3 | 15 | 3.46 |
| 31 | RPL22 | P35268 | 3 | 15 | 2.88 |
| 32 | USP10 | Q14694 | 3 | 87 | 1.60 |
| 33 | PTPN14 | Q6PJC3 | 3 | 160 | 2.59 |
| 34 | RPL5 | P46777 | 2 | 34 | 3.12 |
| 35 | NONO | Q15233 | 2 | 54 | 5.04 |
| 36 | PCBP1 | Q15365 | 2 | 37 | 4.06 |
| 37 | ATP5B | H0YH81 | 2 | 38 | 3.58 |
| 38 | EIF3F | 000303 | 2 | 38 | 1.77 |
| 39 | FAF1 | Q9UNN5 | 1 | 74 | 2.50 |
| 40 | DCTN3 | 075935 | 1 | 21 | 1.64 |
| 41 | HMGB1 | P09429 | 1 | 26 | 4.75 |
| 42 | P3H3 | Q8IVL6 | 1 | 82 | 1.81 |
| 43 | SMG5 | Q9UPR3 | 1 | 114 | 1.70 |
